# A Miniature On-Chip Methane Sensor Based on an Ultra-Low Loss Waveguide and a Micro-Ring Resonator Filter

**DOI:** 10.3390/mi8050160

**Published:** 2017-05-17

**Authors:** Yingying Qiao, Jifang Tao, Chia-Hung Chen, Jifang Qiu, Ye Tian, Xiaobin Hong, Jian Wu

**Affiliations:** 1 State Key Lab of Information Photonics and Optical Communications, Beijing University of Posts and Telecommunications, Beijing 100876, China; qiaoyingy8646@bupt.edu.cn (Y.Q.); jifangqiu@bupt.edu.cn (J.Q.); tianye8801@bupt.edu.cn (Y.T.); xbhong@bupt.edu.cn (X.H.); 2 Department of Biomedical Engineering, National University of Singapore, Block E4, #04-08, 4 Engineering Drive 3, Singapore 117583, Singapore; biecch@nus.edu.sg; 3 Institute of Microelectronics, A*STAR, 11 Science Park Road, Science Park II, Singapore 117685, Singapore; taojf@ime.a-star.edu.sg

**Keywords:** waveguides, photonics sensor, gas detection, optical MEMS

## Abstract

A miniature methane sensor composed of a long ultra-low loss waveguide and a micro-ring resonator filter is proposed with high sensitivity and good selectivity. This sensor takes advantage of the evanescent field to implement methane concentration detection at a near infrared band (1650 nm). In the sensor, two waveguides, a strip waveguide and a slot waveguide, are specially designed and discussed based on three common semiconductor materials, including silica, silicon nitride, and silicon. Through simulations and numerical calculations, we determine that for the strip waveguide, the optimal evanescent field ratio (EFR) is approximately 39.8%, while the resolution is 32.1 ppb using a 15-cm waveguide length. For the slot waveguide, the optimal EFR is approximately 61.6%, and the resolution is 20.8 ppb with a 15-cm waveguide length.

## 1. Introduction

Methane is explosive, odorless, colorless, and flammable. It is also considered a greenhouse gas, along with carbon dioxide [1]. Although the concentration of methane in the atmosphere is much lower than that of carbon dioxide, on a per molecule basis, methane warming influence is 72 times more than that of carbon dioxide [2]. Additionally, a mixture of methane and air is extremely explosive when the concentration of methane is approximately 5–15%. Thus, its accumulation in an enclosed environment can have dire consequences. Therefore, the detection of methane is extremely important for safety reasons, especially in areas such as oil and gas refineries, water treatment plants and landfill sites [3].

A number of methods have been previously proposed to detect gas concentration and species. In essence, gas detection methods can be divided into two types: optical and non-optical method. Hodgkinson [4] further divided the optical method into five categories, including non-dispersive infrared (NDIR), spectrophotometry, cavity ring-down spectroscopy (CRDS), tunable diode laser absorption spectroscopy (TDLAS), and photoacoustic spectroscopy (PAS). Alternatively, the non-optical method includes electrochemistry, gas chromatography, carrier catalytic combustion, etc. Both practical applications and advantages determine the chosen method. The detection of methane is a well-studied field. For example, Massie [3] proposed a NDIR method which achieved a sensitivity of up 0.2% lower explosion limit (LEL) (100 ppm); Kim [5] utilized mid-infrared range Fourier transform infrared spectrophotometry based on a hollow core optical fiber, and could achieve a resolution of up to 512 ppb; Tao [6] proposed an evanescent fiber sensor for the detection of methane, and it could detect methane at levels of 0.1% (equal to 1000 ppm); Vuong [7] used nickel oxide as a catalyst, and their proposed sensor yielded a response of 63.5% per 100 ppm methane gas at a working temperature of 400 °C. While all these sensors could achieve monitoring requirements, the drawbacks of some of these sensors included bulky systems, high complexity of use, high cost, high power consumption, sensitive to the environment, and high operation temperature. As such, the urgent need is put forward to fabricate miniature, low power consumption, robust systems.

Recently, integrated photonic solutions are gaining popularity as a promising tool for gas detection. In 2011, Lai [8] proposed an on-chip methane sensor based on a photonic crystal (PC) slot waveguide which used near-IR absorption to detect methane concentration. The proposed sensor was able to detect methane concentrations as low as 100 ppm using a 300-µm long silicon PC slot waveguide, with power consumption at less than 20 mW. Siebert [9,10] previously established the working principles behind infrared integrated optical evanescent field sensors for gas analysis, which we based our sensor design and fabrication on. In the infrared region of the spectrum, most gases exhibit narrow absorption lines, which can be measured at high resolution. Near IR spectra are typically overtones of fundamental vibrations in the mid IR and hence are weaker; for example, it is approximately 100 times weaker for methane [1]. Fortunately, in the near-IR region (especially from 1500 nm to 1650 nm) both light sources and detectors have been well developed with low cost and superior performance. Additionally, many silicon photonics with passive structures (e.g., photonics waveguides) and integration technologies (e.g., flip-chip laser diode bonding) can be used in this band. Based on the above mentioned advantages, gas detection with high accuracy and compact configuration in the near-IR region is ideal.

The objective of this paper is to design a novel, near-IR methane sensor with cheap, simple, and easily-integrated features. This sensor takes full advantage of active and passive components in the near-IR region to achieve ultra-high sensitive methane detection on a small integrated silicon chip. In this paper, two waveguides, a strip waveguide and a slot waveguide, are specially designed and discussed. Through simulations and numerical calculations, the highest achievable resolution is 20.8 ppb for the slot waveguide using a 15-cm waveguide length.

## 2. Theoretical Description

**Figure 1:**
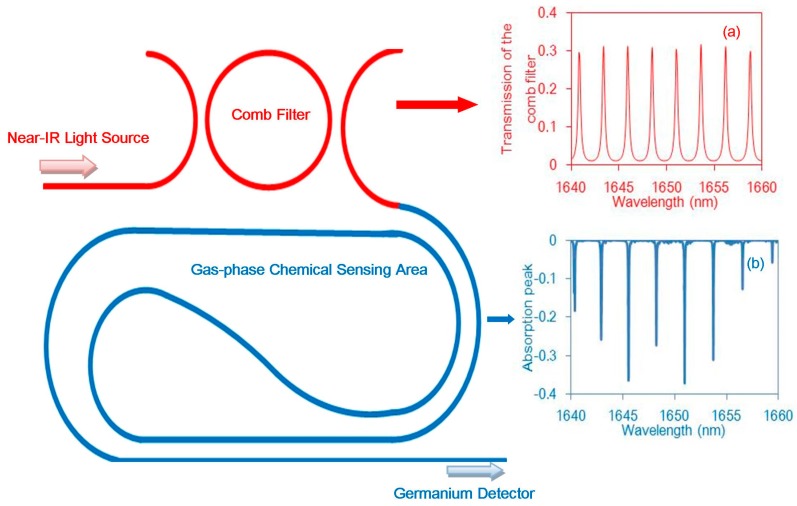
Schematic of the methane sensor and the transmission of the comb filter: transmission spectrum of the comb filter shown in insert (**a**); the methane gas absorption peak is shown in insert (**b**).

The proposed sensor comprises four simple components; a low-cost broadband light source (e.g., light-emitting diode (LED) or amplified spontaneous emission (ASE) chips), a micro-ring resonator filter, a normal waveguide, and a low-noise photodiode, as shown in Figure 1. Based on its working principle, two aspects are discussed: the first is the micro-ring resonator comb filter, and the second is the long low-loss waveguide around which light and methane gas interact with each other.

As methane has several absorption lines in the 1650 nm band, the light source used is a low-cost and well-developed near-IR broadband light source which can cover many absorption lines of methane gas. Unfortunately, many other gases also have absorption lines located in this waveband. To overcome this obstacle, a micro-ring resonator acting as comb filter is designed to match the methane absorption lines as many as possible and to avoid cross-gas interference effectively. Light and methane interact with each other in the gas-phase chemical sensing area, and finally the absorbed light is monitored by a high-performance germanium detector. The total working process is shown in Figure 1, the insert (a) represents transmission resonant peaks of the designed micro-ring filter, and the absorption lines of methane are indicated in insert (b). The perfect matching degree between transmission peaks and absorption lines directly determines the sensitivity and selectivity of the designed sensor [11].

For a typical micro-ring resonator, the resonant wavelength equal to the transmission wavelength can be calculated as seen in Equation (1). Here, l is the optical length of the micro-ring, and equal to 2πr ( r is the micro-ring radius), $n_{\mathrm{eff}}$ is the micro-ring effective refractive index, while $\lambda_{\mathrm{res}}$ is the resonance wavelength of the micro-ring.


(1) $$\lambda_{\mathrm{res}}=\frac{n_{\mathrm{eff}}l}{m},\;m=1,2,3\ldots$$

From Equation (1), it can be seen that if the wavelength of the light fits the multiple of an integer of the wavelength, the micro-ring will be in resonance [12]. If the incident wavelength satisfies the resonance condition, the light with wavelength $\lambda_{\mathrm{res}}$ will be enhanced and others will be suppressed [13]. Finally, only $\lambda_{\mathrm{res}}$ can drop from the output port. Free spectral range (FSR) is defined as the space between two adjacent transmission peaks, and it can be calculated by the following equation:

(2) $$\mathrm{FSR}=\frac{\lambda_{\mathrm{res}}{}^{2}}{n_{g}l}$$

For FSR design, by matching transmission peaks to the methane absorption lines, the sensor will have good sensitivity and selectivity.

In this paper, the proposed methane sensor uses the principle of evanescent field absorption as its mode of operation.

Evanescent field sensors have been intensely researched for several decades, with applications in many areas. The principle of these sensors is based on total internal reflection at the interface between two media with different refraction indexes [14]. When the incident angle is above the critical angle, light travelling through the high refractive index waveguide undergoes total internal reflection and most of the energy is confined within the high refractive index waveguide core. However, there will be an evanescent field extending to the cladding region formed by the surrounding media [15].

The evanescent field, which can extend to the surrounding media, will interact with gas and be absorbed if the wavelength range of the light source can cover the absorption lines of the gas. Then, the intensity of the absorbed light depends on the concentration of the gas. This can be described by the Beer-Lambert Law [9]:

(3) $$I\left(\lambda \right)=I_{o}\left(\lambda \right)\exp\left(-\eta \varepsilon_{\lambda }cl-\alpha_{\mathrm{base}}l\right)$$

where $I_{o}\left(\lambda \right)$ is the output optical power without the target gas, I(λ) is the output optical power with the target gas, c is the concentration of the target gas, $\varepsilon_{\lambda }$ is the absorption coefficient of the gas at wavelength λ (for methane, the range of $\varepsilon_{\lambda }$ is from 0.035 cm^−1^ to 0.3 cm^−1^ in 1650 nm band) [11], l is the length of the optical path, $\alpha_{\mathrm{base}}$ is the waveguide loss, and η is the evanescent field ratio (EFR), which can be calculated by the following expression [15]:

(4) $$\eta =I_{\mathrm{gas}}/I_{\mathrm{all}}=\iint_{\mathrm{gas}}\vec{S}\cdot \vec{n}dxdy÷\iint_{\mathrm{all}}\vec{S}\cdot \vec{n}dxdy$$

where $\vec{S}$ is the Poynting Vector of the mode field in the waveguide, and $\vec{n}$ is the normal vector to the waveguide cross-section.

The sensitivity of the sensor *S* is considered as the change in optical power with gas concentration, and can be described by the following [15]:

(5) $$S=-\eta \varepsilon_{\lambda }lI_{o}\left(\lambda \right)\exp\left(-\eta \varepsilon_{\lambda }cl-\alpha_{\mathrm{base}}l\right)$$

As we can see, the sensitivity of the methane sensor critically depends on the EFR, and can be increased by maximizing the EFR.

Another very important parameter to describe the performance of the sensor would be resolution ( ΔC ). This denotes the smallest detectable gas concentration. We can calculate it by the following formula [9]:

(6) $$\Delta C=\frac{\mathrm{NEP}\times \sqrt{B}}{\left|S\right|}$$

where NEP means noise equivalent power, which is a parameter of the detector to describe the minimum detectable power. B is the band width of the detector. Thus, the higher the sensitivity, the lower the resolution, and the better performance.

**Figure 2:**
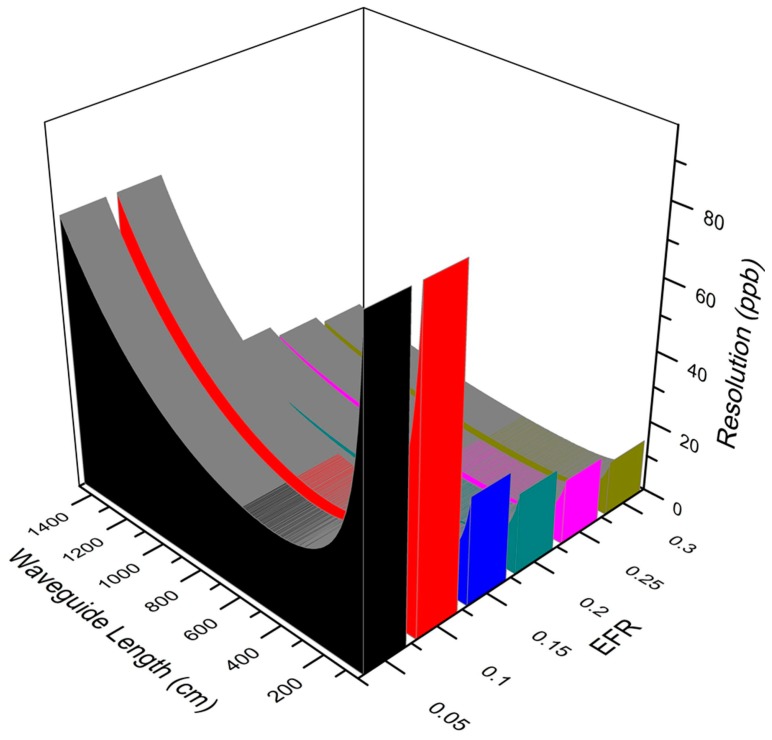
A 3D graph of the variation trends of resolution with waveguide lengthening with different evanescent field ratios (EFRs): the vertical axis represents resolution, the colorized curve volumes represent different EFRs. A clear relation between waveguide length, EFRs, and resolution is shown.

In practical applications, resolution is usually used to judge the performance of a sensor and is especially paid attention to by most consumers. By combining Equations (5) and (6), a relationship between resolution, length of sensing area and different EFRs can be obtained, as seen in Figure 2.

From Figure 2, all curves have a dip with waveguide lengthening, which indicates that an optimal length exists to achieve the best resolution. This numerical analysis thus offers a significant theoretical guide in the design process of a sensor.

## 3. Simulation and Results

**Figure 3:**
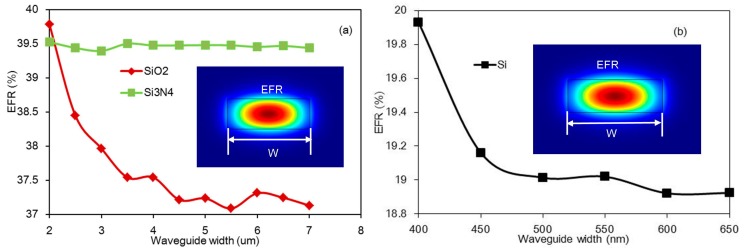
The evanescent field ratio (EFR) of the strip waveguide versus waveguide width with different materials: (**a**) the waveguide fabricated by silica (red line) and silicon nitride (green line); (**b**) the waveguide fabricated by silicon.

**Figure 4:**
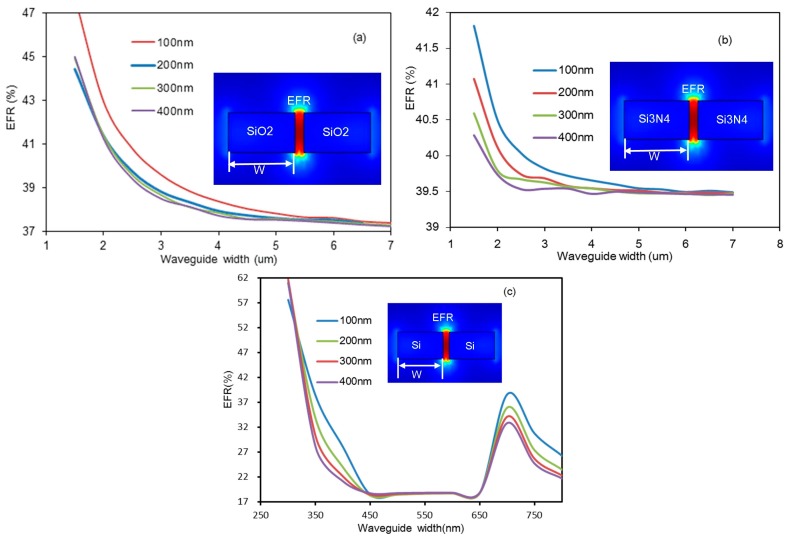
Evanescent field ratio versus waveguide width with different slot region widths for silica, silicon nitride, and silicon waveguides: (**a**) silica waveguide EFR; (**b**) silicon nitride waveguide EFR; (**c**) silicon waveguide EFR.

This report introduces two different waveguide structures and analyzes their performance. One is long strip waveguide cladding by methane gas, the other is a slot waveguide surrounded by methane, as the inserts show in Figure 3 and Figure 4, respectively. The gas will interact with the evanescent field around the waveguide, so our design should ensure that more evanescent field power extends to the gas to enhance the interaction between gas and light. In the sensor, all waveguides are discussed based on three common semiconductor materials; these are: silica, silicon nitride, and silicon. The refractive indexes of the three materials are different, thus according to theoretical analysis, the higher the refractive index of strip waveguide, the stronger the restriction of light, which causes the EFR to decrease. However, for the slot waveguides, the light is confined within the slot region, so the higher the refractive index, the higher the EFR.

In the design process, we first designed waveguide dimensions to ensure the proper guiding of light through it when the wavelength is 1653 nm. Following which, the EFR value was investigated at different waveguide widths. For each material of the waveguide, this paper analyzes a single height: the height of the silicon and silicon nitride waveguides is 220 nm, while the height of the silica waveguide is 400 nm. Since the refractive index of silica is less than others, it requires a larger height to support the guiding mode. For the long strip waveguide, when the waveguide is fabricated by silica or silicon nitride, in order to support the single mode, the sweeping width region is set to 2 μm–7 μm. However, for the higher refractive index silicon waveguide, the selected width region is 400 nm–650 nm, because it cannot support the guiding mode when the width is smaller than 400 nm, and there will be multimode transmission when the width grows larger than 700 nm. The mode analysis is performed by the finite element method. Given the long waveguide dimensions, we chose the beam envelope interface of a commercial software, COMSOL, to implement the boundary mode analysis. The calculation results are shown in Figure 3.

The simulation results of the silica and silicon nitride strip waveguides are shown in Figure 3a. When the waveguide broadens, the evanescent field ratio of the silicon nitride waveguide changes slightly, as indicated by the green line shown in Figure 3a, and there is a limit on the width of the silicon nitride waveguide. When the width is too small, the mode becomes leaky and is no longer supported as a guiding mode. When the waveguide width is approximately 2 μm, the evanescent field ratio has the maximum value, at which the EFR is 39.5%. For the silica waveguide, as indicated by the red line in Figure 3a, when waveguide width widened, the EFR decreases sharply because most of the field is confined in the waveguide core. The maximum EFR is 39.8% using a 2 μm width. For the silicon waveguide, the simulation results of which are shown in Figure 3b, in order to ensure single mode transmission, the chosen width region is 400–650 nm, and the maximum EFR is 20% when the width is 400 nm. This maximum value is much smaller than that of the silica and silicon nitride waveguides because the silicon waveguide’s larger refractive index causes more light to be confined within the waveguide core.

For the slot waveguide structure, during simulation, the selected width region and height of the three materials’ waveguides are same as the strip waveguide. The results of the simulation are shown in Figure 4. For the silica waveguide, the results are shown in Figure 4a. It is observed that when the width is less than 1 μm, the evanescent field ratio sharply increases. The reason for the observed trend is that at widths less than 1 μm, the width is too small to support the guiding mode and the mode becomes leaky at the slot region. When the slot region is 100 nm and the waveguide width is 1.5 μm, the EFR is maximized, and the optimal value is approximately 42.9%. In addition, the width of the slot region has little impact on the EFR, but the waveguide width has a big impact on the EFR. The results of the silicon nitride waveguide can be seen in Figure 4b. When the slot region is 100 nm and the waveguide width is 1.5 μm, the maximal EFR is 41.8%. Clearly, when the waveguide is narrow, the smaller the slot region, and consequently, the higher the EFR. With waveguide widening, the slot region gradually has little impact on the EFR. The results of the silicon waveguide simulation are detailed in Figure 4c. With the increase in waveguide width, the EFR first decreases and then increases when the width becomes larger than 700 nm, because the waveguide is in multimode transmission. Finally, the maximal EFR is approximately 61.6% when the slot region is 300 nm and the waveguide width is 300 nm.

## 4. Analysis and Calculation

High sensitivity and low resolution are the most important criteria for evaluating a sensor. As such, we first need to calculate the sensor’s sensitivity and resolution to evaluate its performance.

The sensor consists of a long optical path to enhance the interaction between light and the target gas, so we need to calculate an optimal length range. Methane has several strong absorption lines in the 1650 nm band, and from this band we selected 1653 nm as the wavelength to implement our study. As an example, we calculated the sensing resolution and sensitivity of methane in the atmosphere using parameters from near-IR detectors and low-cost broadband light sources. Incident light sources have been developed for many years, and recently they can provide large optical powers at more than 100 mW with approximately 5% of the optical power included in our investigative band. In addition, the NEP of near-IR detectors is often three orders less than mid-IR detectors. H. Mohseni [16] developed a detector in which the NEP could decrease to 2 × 10^−16^ W/Hz^1/2^. The base concentration of methane was chosen to be the approximate atmospheric level of 2 ppm; the absorption coefficient of methane is 0.3 cm^−1^ [15]; coupling loss is the most important additional loss and is approximately 2 dB. Ultra-low-loss waveguides have been successfully manufactured previously [17,18], and the lowest waveguide loss has been reported to be 1.2 dB/m. In this report, the waveguide loss is set 0.01 dB/cm.

Under the above mentioned simulation conditions, from Equations (5) and (6), we can calculate the relationship between the waveguide length and the sensitivity and resolution of the sensor. Because of the ultra-low waveguide loss, the optimal length we calculated is approximately 435 cm. While the optimal waveguide length is so long, it is undesirable because it forces our proposed sensor design to be quite large. In reality, short waveguide lengths can still provide exceptional performances and satisfy many applications. As an example, the analysis results using a 15-cm waveguide length are shown in Table 1.

For slot waveguides, after implementing the above calculation steps, the results are better than that of the strip waveguide, because of the higher EFR. This is especially true for the silicon waveguide, which has a high refractive index that causes light to be tightly restricted in a slot region, making the EFR much higher than that of the silica and silicon nitride waveguides. At a 15-cm wavelength guide length, the simulation and calculated results are shown in Table 2.

Last but not least, the high step in refractive index between the waveguide and the surrounding gas allows for very small bending radii. Thus, a long waveguide can be realized on small chip area by using a spiral waveguide design. Several simple samples have been proposed [9,10]. Given the state-of-the-act technology, it is entirely feasible to fabricate this sensor.

## 5. Conclusions

In conclusion, the report proposes a type of miniature sensor structure composed of a micro-ring resonator and a long low loss waveguide without a gas cell. The focal point of the report is about the design of the two different low loss waveguides. As an example, we analyze the sensitivity and resolution of methane sensors that uses the following materials: silica, silicon nitride, silicon. According to simulations and reference data, for the strip waveguide, the maximal EFR is 39.8% when the sensor is made of silica. In addition, the sensitivity is 8.7 nW/ppm, and the resolution is 32.1 ppb when the waveguide length is 15 cm for methane gas. For the slot waveguide, the results indicate a better performance than that of the strip waveguide; the maximal EFR is 61.1% when the sensor made of silicon, while the sensitivity is 13.4 nW/ppm and the resolution is 20.8 ppb using a 15-cm waveguide length. The knowledge gained from this investigation provides a good basis for the development of portable sensors. In addition, the 1650 nm band includes many other kinds of gas absorption lines, so the sensor’s working principle can be adapted for sensing other types of gases.

## Figures and Tables

**Table 1:** The analysis results of a strip waveguide.

Material	Height (nm)	Width (μm)	EFR	Sensitivity (nw/ppm)	Resolution (ppb)
SiO_2_	400	2	39.8%	8.7	32.1
Si	220	0.4	20%	4.3	64
Si_3_N_4_	400	2	39.5%	8.6	32.4

**Table 2:** The analysis results of a slot waveguide.

Material	Height (nm)	Width (μm)	Slot (nm)	EFR	Sensitivity (nw/ppm)	Resolution (ppb)
SiO_2_	400	1.5	100	42.9%	9.3	29.8
Si	220	0.3	300	61.6%	13.4	20.8
Si_3_N_4_	400	1.5	100	41.8%	9.1	30.6
